# Soft tissue radiodensity parameters mediate the relationship between self-reported physical activity and lower extremity function in AGES-Reykjavík participants

**DOI:** 10.1038/s41598-021-99699-7

**Published:** 2021-10-11

**Authors:** Kyle J. Edmunds, Ozioma C. Okonkwo, Sigurdur Sigurdsson, Sarah R. Lose, Vilmundur Gudnason, Ugo Carraro, Paolo Gargiulo

**Affiliations:** 1 Department of Medicine, University of Wisconsin School of Medicine and Public Health, Madison, WI USA; 2 Wisconsin Alzheimer’s Disease Research Center, University of Wisconsin School of Medicine and Public Health, Madison, WI USA; 3 Wisconsin Alzheimer’s Institute, University of Wisconsin School of Medicine and Public Health, Madison, WI USA; 4 Institute for Biomedical and Neural Engineering, Reykjavík University, Reykjavík, Iceland; 5 Icelandic Heart Association (Hjartavernd), Kópavogur, Iceland; 6 Faculty of Medicine, University of Iceland, Reykjavík, Iceland; 7 Department of Biomedical Sciences, University of Padova, Padua, Italy; 8 Department of Science, Landspítali, Reykjavík, Iceland

**Keywords:** Biomedical engineering, Computational models, Ageing

## Abstract

Although previous studies have highlighted the association between physical activity and lower extremity function (LEF) in elderly individuals, the mechanisms underlying this relationship remain debated. Our recent work has recognized the utility of nonlinear trimodal regression analysis (NTRA) parameters in characterizing changes in soft tissue radiodensity as a quantitative construct for sarcopenia in the longitudinal, population-based cohort of the AGES-Reykjavík study. For the present work, we assembled a series of prospective multivariate regression models to interrogate whether NTRA parameters mediate the 5-year longitudinal relationship between physical activity and LEF in AGES-Reykjavík participants. Healthy elderly volunteers from the AGES-Reykjavík cohort underwent mid-thigh X-ray CT scans along with a four-part battery of LEF tasks: normal gait speed, fastest-comfortable gait speed, isometric leg strength, and timed up-and-go. These data were recorded at two study timepoints which were separated by approximately 5 years: AGES-I (*n* = 3157) and AGES-II (*n* = 3098). Participants in AGES-I were likewise administered a survey to approximate their weekly frequency of engaging in moderate-to-vigorous physical activity (PA_AGES-I_). Using a multivariate mediation analysis framework, linear regression models were assembled to test whether NTRA parameters mediated the longitudinal relationship between PA_AGES-I_ and LEF_AGES-II_; all models were covariate-adjusted for age, sex, BMI, and baseline LEF, and results were corrected for multiple statistical comparisons. Our first series of models confirmed that all four LEF tasks were significantly related to PA_AGES-I_; next, modelling the relationship between PA_AGES-I_ and NTRA_AGES-II_ identified muscle amplitude (*N*_*m*_) and location (*μ*_*m*_) as potential mediators of LEF to test. Finally, adding these two parameters into our PA_AGES-I_ → LEF_AGES-II_ models attenuated the prior effect of PA_AGES-I_; bootstrapping confirmed *N*_*m*_ and *μ*_*m*_ as significant partial mediators of the PA_AGES-I_ → LEF_AGES-II_ relationship, with the strongest effect found in isometric leg strength. This work describes a novel approach toward clarifying the mechanisms that underly the relationship between physical activity and LEF in aging individuals. Identifying *N*_*m*_ and *μ*_*m*_ as significant partial mediators of this relationship provides strong evidence that physical activity protects aging mobility through the preservation of both lean tissue quantity and quality.

## Introduction

Sarcopenia is consistently identified in literature as an independent risk factor for a host of deleterious health outcomes in the elderly^1–5^. Although clinical definitions of the syndrome remain debated, there is growing consensus that sarcopenia is characterized primarily by the age-related loss of skeletal muscle strength and function, followed by the commensurate loss of lean tissue mass^6–8^. The loss of mobility incurred by these characteristic changes has been directly associated with increased risk for injurious falls, physical frailty, disability, and eventual mortality^9^, and a growing body of evidence supports the relationship between sarcopenia and other aging-related diseases or comorbidities. For example, decreased muscle strength and mass have been found to progress alongside advancing stages of aging-related cognitive decline^10^, Alzheimer’s disease (AD) dementia^11,12^, and brain atrophy in early preclinical AD^13^. Characteristic inflammatory cytokine production and myosteatosis in sarcopenia have likewise been linked with the onset of type-2 diabetes^14^, hypertension^15^, dyslipidemia^16^, coronary heart disease (CHD)^17^, and all-type cardiovascular disease (CVD)^18–21^. Taken together, these findings suggest that sarcopenia’s etiology or pathophysiology is entangled with those of other age-related diseases—a notion further reflected by sarcopenia’s high and growing global prevalence^22^.

Physical activity may play a critical role in not only protecting against muscle degeneration and mobility loss from sarcopenia, but in attenuating its downstream effects on other health outcomes^22^. Indeed, the many advantages of maintaining an active lifestyle throughout one’s lifecourse have been repeatedly demonstrated in literature. Regarding direct effects on skeletal muscle health, resistance exercise training in elderly individuals has been correlated with greater muscle strength, increased lean mass, and improved myofibrillar protein accretion^23^. Aerobic exercise has also been shown to improve insulin sensitivity^24^ and protect against a host of metabolic, cardiovascular, and oncological diseases^9^. A growing body of literature further cites physical activity as an important protective factor against AD pathogenesis by promoting brain plasticity and neurotrophic factors^25^, improving cerebral glucose metabolism^26^, and reducing cerebrospinal fluid AD biomarkers^27^.

To interrogate the role of skeletal muscle health on the relationship between physical activity and mobility necessitates the development of standardized quantitative methods for assessing and monitoring soft tissue changes in aging individuals. In this regard, recent investigations have realized the potential of X-ray computed tomography (CT) image analysis to quantify changes in muscle volume and quality^28,29^. This is performed by the linear transformation of radiodensitometric absorption values, represented in Hounsfield units (HU), which can then be used to illustrate changes in muscle mass through segmented cross-sectional areas^30–34^, while average HU values can be used as a construct for muscle quality^29,35,36^. Our team has recently extended this literature through the computational modelling of entire radiodensitometric distributions from CT cross-sections of the upper thigh, featuring the novel nonlinear trimodal regression analysis (NTRA) method^37,38^. This method results in eleven subject-specific distribution parameters that altogether present a unique index for skeletal muscle health and have shown differential sensitivity to nutritional factors, lower extremity function (LEF), cardiovascular pathophysiology, and type-2 diabetes mellitus in healthy aging adults from the longitudinal AGES-Reykjavík study^38–40^.

**Figure 1:**
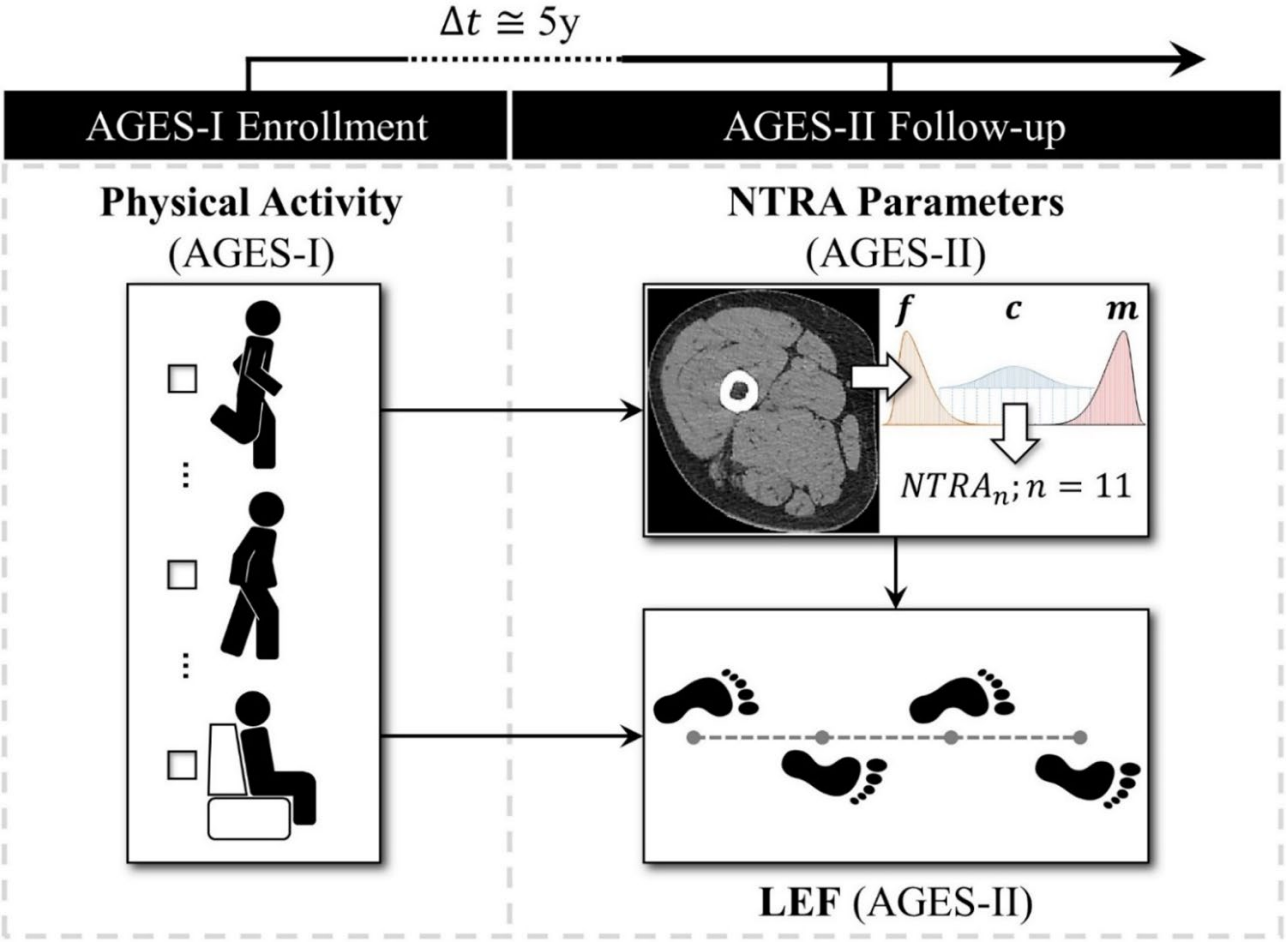
Graphical summary of the present NTRA-based multiple mediation study, showing the theorized role of soft tissue radiodensity changes (NTRA parameters) on the 5-year longitudinal relationship between self-reported physical activity and LEF in elderly AGES-Reykjavík subjects.

While these works have highlighted the potential of NTRA parameters in quantitatively describing sarcopenia and its association with aging health, the mediating role of NTRA-based soft tissue radiodensity on the relationship between self-reported physical activity and LEF was not investigated. This was the primary motivation for the present work, where NTRA mediation is interrogated using prospective multivariate regression models of LEF performance constructed from longitudinal AGES-Reykjavík data (Fig. 1).

## Results

### AGES-Reykjavik dataset covariates, LEF tasks, and NTRA parameters

Prior to the construction of multivariate regression models for the present mediation analysis, descriptive statistics, mean LEF task performance, and mean NTRA parameters from mid-thigh CT scans were assembled from AGES-I and AGES-II data, and pairwise $t$ tests were performed between datasets to illustrate 5-year longitudinal changes. Table 1 summarizes these results.

**Table 1:** AGES-I and AGES-II covariate statistics, LEF tasks, and NTRA parameters.

Variable	AGES-I: $n=$ 3157	AGES-II: $n=$ 3098	
Age (years), mean (SD)	74.9 (4.8)	80.0 (4.8)^a^	
Women, *n* (%)	1832 (58.0)	1807 (58.3)	
BMI (kg/m^2^), mean (SD)	27.3 (4.2)	26.8 (4.4)	
Underweight ($<$ 18.5 kg/m^2^), *n* (%)	18 (0.6)	37 (1.2)	
Normal (18.5–25 kg/m^2^), *n* (%)	949 (30.1)	1090 (35.2)	
Overweight (25–30 kg/m^2^), *n* (%)	1454 (46.1)	1309 (42.3)	
Obese ($>$ 30 kg/m^2^), *n* (%)	736 (23.3)	662 (21.4)	
PA_AGES-I_, mean score (SD)	2.6 (1.6)	N/A	
(1) ‘*Never*’, *n* (%)	1258 (39.8)		
(2) ‘*Rarely*’, *n* (%)	527 (16.7)		
(3) ‘*Occasionally*’, *n* (%)	243 (7.7)		
(4) ‘*Moderate*’, *n* (%)	578 (18.3)		
(5) ‘*High*’, *n* (%)	551 (17.5)		

LEF task	AGES-I	AGES-II	
Fast gait speed (GSF; m/s), mean (SD)	1.34 (0.25)	1.26 (0.26)^a^	
Normal gait speed (GSN; m/s), mean (SD)	0.99 (0.19)	0.92 (0.20)^a^	
Isometric leg strength (STR; N), mean (SD)	337.9 (115.9)	279.5 (101.4)^a^	
Timed up-and-go (TUG; s), mean (SD)	11.5 (2.7)	12.7 (3.8)^a^	

Tissue	NTRA parameter	AGES-I	AGES-II
Fat	Amplitude: $N$, mean (SD)	62.0 (33.1)	62.3 (33.2)
	Location: $\mu$, mean (SD)	− 117.8 (3.3)	− 117.2 (4.7)^a^
	Width: $\sigma$, mean (SD)	8.2 (6.0)	8.1 (5.7)
	Skewness: $\alpha$, mean (SD)	− 2.5 (2.1)	− 2.5 (2.0)
Conn	Amplitude: $N$, mean (SD)	41.6 (8.4)	41.8 (9.3)
	Location: $\mu$, mean (SD)	− 24.1 (28.6)	− 25.9 (28.2)^a^
	Width: $\sigma$, mean (SD)	25.1 (5.7)	24.6 (5.6)^a^
Muscle	Amplitude: $N$, mean (SD)	78.0 (17.9)	72.6 (17.4)^a^
	Location: $\mu$, mean (SD)	61.5 (2.7)	60.9 (2.9)^a^
	Width: $\sigma$, mean (SD)	8.6 (2.2)	9.2 (2.6)^a^
	Skewness: $\alpha$, mean (SD)	2.8 (0.8)	3.0 (0.8)^a^

^a^Significant difference ($\mathrm{P}<$ 0.05) between AGES-I and AGES-II.

As shown, from the AGES-I sample size of $n=$ 3157, $n=$ 3098 of these individuals remained as full participants in AGES-II. Aside from age, there were no significant differences between AGES-I and AGES-II covariates, but LEF task performance was significantly worse in AGES-II across all four tasks, suggesting a generalized loss of mobility in the cohort. Significant between-dataset changes in mid-thigh NTRA parameters likewise describe a general radiodensitometric shift toward characteristic sarcopenia, illustrated primarily in lean muscle; in AGES-II, subjects’ muscle distributions were significantly wider and lower in amplitude, with an increase in positive skewness and a shift in location toward 0 HU. This location shift also occurred in fat, while loose connective tissue distributions were significantly wider with a more negative mean location.

### NTRA parameter mediation analysis on the relationship between PA_AGES-I_ and LEF_AGES-II_

As the first step in mediation analysis, multivariate multiple regression models were assembled on the longitudinal relationship between physical activity recorded at AGES-I (PA_AGES-I_) and LEF performance recorded at AGES-II, approximately 5 years later (LEF_AGES-II_). Model covariates were age, sex, BMI, and baseline LEF_AGES-I_ defined respectively by each of the four LEF tasks. Table 2 shows these results.

**Table 2:** Multivariate multiple regression models of the relationship between PA_AGES-I_ and LEF_AGES-II_.

Variable	Fast gait speed (GSF)			Normal gait speed (GSN)			Isometric leg strength (STR)			Timed up-and-go (TUG)		
	β	SE	p-value	β	SE	p-value	β	SE	p-value	β	SE	p-value
PA_AGES-I_	0.1686	0.003	< 0.001	0.1755	0.002	< 0.001	0.1060	0.934	< 0.001	− 0.1406	0.038	< 0.001
Age	− 0.3454	0.001	< 0.001	− 0.4046	0.001	< 0.001	− 0.2603	0.314	< 0.001	0.3467	0.014	< 0.001
Sex	− 0.3032	0.009	< 0.001	− 0.1619	0.007	< 0.001	− 0.5895	2.967	< 0.001	0.0497	0.130	0.003
BMI	− 0.1292	0.001	< 0.001	− 0.1466	0.001	< 0.001	0.0889	0.342	< 0.001	0.1454	0.015	< 0.001
LEF_AGES-I_	0.0180	0.018	0.279	0.0154	0.017	0.335	0.0094	0.013	0.512	0.0180	0.023	0.285

All four PA_AGES-I_ hypothesis tests surpassed their respective thresholds for Holm–Bonferroni (HB) correction.

As shown in Table 2, all four PA_AGES-I_ β coefficients were significantly related to LEF_AGES-II_ ($p<$ 0.05). Furthermore, all four coefficients were less than their ranked thresholds for HB correction, suggesting that all four LEF_AGES-II_ tasks were significantly related to PA_AGES-I_, even after correcting for multiple comparisons.

### The relationship between PA_AGES-I_ and hypothesized NTRA_AGES-II_ mediators

Next, the relationship between PA_AGES-I_ and hypothesized NTRA_AGES-II_ mediators was tested, again using multivariate multiple regression modelling. Results from these analyses are given in Table 3.

**Table 3:** Multivariate multiple regression models of the relationship between PA_AGES-I_ and hypothesized NTRA_AGES-II_ mediators.

Tiss	Variable	Amplitude: $N$			Location: $\mu$			Width: $\sigma$			Skewness: $\alpha$			
		β	SE	p-value	β	SE	p-value	β	SE	p-value	β		SE	p-value
*Fat*	PA_AGES-I_	− 0.0164	0.214	0.108	− 0.0233	0.054	0.203	0.0409	0.058	0.011^†^	− 0.0323		0.021	0.054
	Age	− 0.0165	0.070	0.107	0.0320	0.018	0.081	0.0354	0.019	0.028	− 0.0703		0.007	< 0.001
	Sex	0.5544	0.676	< 0.001	0.0476	0.170	0.008^†^	− 0.4222	0.184	< 0.001	0.2820		0.066	< 0.001
	BMI	0.5884	0.077	< 0.001	0.0761	0.020	< 0.001	− 0.2000	0.021	< 0.001	0.2587		0.008	< 0.001
*Muscle*	PA_AGES-I_	0.0841	0.130	< 0.001	0.1076	0.031	< 0.001	− 0.0408	0.025	0.008^†^	− 0.0276		0.009	0.114
	Age	− 0.2152	0.043	< 0.001	− 0.2434	0.010	< 0.001	0.1381	0.008	< 0.001	0.1822		0.003	< 0.001
	Sex	− 0.5082	0.410	< 0.001	− 0.0717	0.099	< 0.001	0.2243	0.081	< 0.001	0.1920		0.028	< 0.001
	BMI	0.5135	0.047	< 0.001	0.1242	0.011	< 0.001	0.4722	0.009	< 0.001	0.1689		0.003	< 0.001
*Connective*	PA_AGES-I_	− 0.0001	0.071	0.991	0.0243	0.224	0.054	0.0155	0.063	0.384		N/A		
	Age	− 0.0437	0.023	< 0.001	− 0.0392	0.074	0.002	− 0.1437	0.021	< 0.001				
	Sex	− 0.3507	0.225	< 0.001	− 0.6422	0.709	< 0.001	0.1876	0.198	< 0.001				
	BMI	0.6716	0.026	< 0.001	− 0.3107	0.081	< 0.001	− 0.0581	0.022	0.001				

^†^p-value did not surpass HB correction for multiple comparisons.

After controlling for multiple comparisons, PA_AGES-I_ was found to be a significant predictor ($p<$ 0.05) for muscle amplitude (${N}_{m}$) and location (${\mu }_{m}$), suggesting their potential to serve as mediators of the relationship between PA_AGES-I_ and LEF_AGES-II_.

### The relationship between LEF_AGES-II_ and NTRA_AGES-II_ parameters significantly related to PA_AGES-I_

The penultimate step in mediation analysis involved testing the relationship between LEF_AGES-II_ and ${N}_{m}$ and ${\mu }_{m}$ as the two NTRA_AGES-II_ parameters found to be significantly related to PA_AGES-I_. Results from these analyses are presented in Table 4.

**Table 4:** Multivariate multiple regression models of the relationship between LEF_AGES-II_ and NTRA_AGES-II_ parameters significantly related to PA_AGES-I_.

Variable	Fast gait speed (GSF)			Normal gait speed (GSN)			Isometric leg strength (STR)			Timed up-and-go (TUG)		
	β	SE	p-value	β	SE	p-value	β	SE	p-value	β	SE	p-value
${N}_{m}$	0.2990	3.9E−4	< 0.001	0.2722	2.9E−4	< 0.001	0.3265	0.126	< 0.001	− 0.0482	0.006	< 0.001
${\mu }_{m}$	0.0983	0.002	< 0.001	0.1226	0.001	< 0.001	0.1023	0.522	< 0.001	− 0.1606	0.024	< 0.001
Age	− 0.2805	0.001	< 0.001	− 0.3385	0.001	< 0.001	− 0.1783	0.318	< 0.001	0.2874	0.014	< 0.001
Sex	− 0.1530	0.011	< 0.001	− 0.0268	0.008	0.184	− 0.4202	3.61	< 0.001	− 0.0615	0.164	0.004
BMI	− 0.3099	0.001	< 0.001	− 0.3148	0.001	< 0.001	− 0.0978	0.408	< 0.001	0.2843	0.019	< 0.001
LEF_AGES-I_	0.0244	0.017	0.136	0.0132	0.017	0.401	0.0092	0.012	0.502	0.0129	0.023	0.437

All eight PA_AGES-I_ hypothesis tests surpassed their respective thresholds for HB correction.

Here, across all four LEF tasks, ${N}_{m}$ and ${\mu }_{m}$ were significant predictors ($p<$ 0.05) even after HB correction for multiple comparisons. These results confirm the relationship between ${N}_{m}$ and ${\mu }_{m}$ and each LEF task, suggesting the particular utility of muscle radiodensity as a quantitative correlate of LEF performance.

### Testing the mediating roles of ${N}_{m}$ and ${\mu }_{m}$

As prior regression models implicated ${N}_{m}$ and ${\mu }_{m}$ as potential NTRA-based mediators of the relationship between PA_AGES-I_ and LEF_AGES-II_, the final step was to test their indirect mediation effects along with any direct effects from PA_AGES-I_ on LEF_AGES-II_. This was performed first by adding ${N}_{m}$ and ${\mu }_{m}$ as mediators to our prior PA_AGES-I_ → LEF_AGES-II_ regression models (see Table 2) and testing the attenuation of PA_AGES-I_ β coefficients. Results from these analyses are shown in Table 5.

**Table 5:** Prior PA_AGES-I_ → LEF_AGES-II_ regression models with ${N}_{m}$ and ${\mu }_{m}$ added as theorized mediators.

	Fast gait speed (GSF)			Normal gait speed (GSN)			Isometric leg strength (STR)			Timed up-and-go (TUG)		
	β	SE	p-value	β	SE	p-value	β	SE	p-value	β	SE	p-value
**Variable**												
PA_AGES-I_	0.1377	0.003	< 0.001	0.1431	0.002	< 0.001	0.0720	0.894	< 0.001	− 0.1118	0.041	< 0.001
${N}_{m}$	0.2781	3.9E−4	< 0.001	0.2494	2.8E−4	< 0.001	0.3162	0.126	< 0.001	− 0.0443	0.006	< 0.001
${\mu }_{m}$	0.0853	0.002	< 0.001	0.1092	0.001	< 0.001	0.0953	0.522	< 0.001	− 0.1464	0.023	< 0.001
Age	− 0.2716	0.001	< 0.001	− 0.3264	0.001	< 0.001	− 0.1718	0.317	< 0.001	0.2779	0.014	< 0.001
Sex	− 0.1522	0.011	< 0.001	− 0.0261	0.008	0.191	− 0.4192	3.60	< 0.001	− 0.0622	0.162	0.003
BMI	− 0.2853	0.001	< 0.001	− 0.2876	0.001	< 0.001	− 0.0848	0.410	< 0.001	0.2630	0.019	< 0.001
LEF_AGES-I_	0.0224	0.017	0.166	0.0135	0.017	0.384	0.0104	0.012	0.443	0.0139	0.023	0.399
**Prior effect**												
PA_AGES-I_	0.1686	0.003	< 0.001	0.1755	0.002	< 0.001	0.1060	0.934	< 0.001	− 0.1406	0.038	< 0.001
*% Change*	− **18.3%**			− **18.5%**			− **32.1%**			− **20.3%**		

All twelve hypothesis tests surpassed their respective thresholds for HB correction.

The addition of ${N}_{m}$ and ${\mu }_{m}$ NTRA parameters attenuated prior PA_AGES-I_ β coefficients for all four LEF tasks. This attenuation effect differed according to task, ranging from − 18.3% (GSF) to − 32.1% (STR). Taken together, ${N}_{m}$ and ${\mu }_{m}$ are significant partial mediators of the 5-year longitudinal relationship between self-reported PA and LEF, with the strongest mediating effect seen in STR performance.

Finally, to further test ${N}_{m}$ and ${\mu }_{m}$ mediation, a bootstrapping procedure using the Hayes and Preacher SPSS macro^41^ was performed to interrogate the significance of both indirect (mediation) effects and direct effects from PA_AGES-I_. Results from this procedure are shown in Table 6.

**Table 6:** Bootstrapped indirect and direct effects analyses for each LEF_AGES-II_ task.

	Fast gait speed (GSF)		Normal gait speed (GSN)		Isometric leg strength (STR)		Timed up-and-go (TUG)	
	Effect	95% CI	Effect	95% CI	Effect	95% CI	Effect	95% CI
**Indirect**								
*Total*_*Ind*_	0.0051	[0.0035, 0.0067]	0.0041	[0.0030, 0.0053]	2.1748	[1.5200, 2.9146]	− 0.0691	[− 0.0905, − 0.0497]
${N}_{m}$	0.0036	[0.0023, 0.0050]	0.0026	[0.0017, 0.0036]	1.5060	[0.9527, 2.1404]	− 0.0398	[− 0.0562, − 0.0255]
${\mu }_{m}$	0.0012	[0.0006, 0.0020]	0.0012	[0.0006, 0.0019]	0.5414	[0.2747, 0.8589]	− 0.0233	[− 0.0366, − 0.0123]
Bootstrap coefficients with 95% CIs								
**Direct**								
PA_AGES-I_	0.0228	[0.0174, 0.0281]	0.0182	[0.0142, 0.0221]	4.6179	[2.8640, 6.3717]	− 0.2676	[− 0.3473, − 0.1878]

95% CI values for all indirect and direct effects did not include zero; therefore, all model effects are significant.

As indicated in Table 6, bootstrapped indirect effects estimates yielded 95% confidence intervals (CI’s) whose range did not encompass zero, or the line of null effect, for any of the four LEF models. Therefore, the indirect effects of PA_AGES-I_ on all four LEF tasks through both ${N}_{m}$ and ${\mu }_{m}$ are all significant.

## Discussion

Prior works have highlighted the quantitative potential of NTRA parameters from mid-thigh CT imaging in associating soft tissue radiodensity changes with aging health outcomes^37–40^. However, this is the first large study to interrogate the mediating role of these radiodensity parameters as an underlying mechanism in the relationship between self-reported physical activity and LEF. This was achieved by assembling prospective multivariate multiple regression models of LEF performance from longitudinal AGES-Reykjavík data, in accordance with an established framework for mediation analysis. From the first step in this procedure, models of the 5-year longitudinal relationship between PA_AGES-I_ and LEF_AGES-II_ indicated that all four LEF tasks were significantly related to PA_AGES-I_ after adjusting for covariates and controlling for multiple statistical comparisons. Next, modelling the relationship between PA_AGES-I_ and NTRA_AGES-II_ parameters as theorized mediators indicated ${N}_{m}$ and ${\mu }_{m}$ as potential mediators of LEF. Finally, adding ${N}_{m}$ and ${\mu }_{m}$ to prior PA_AGES-I_ → LEF_AGES-II_ regression models resulted in the attenuation of PA_AGES-I_ β coefficients, and bootstrapping solidified ${N}_{m}$ and ${\mu }_{m}$ as significant partial mediators.

This work altogether presents a novel approach toward clarifying the nature of the relationship between physical activity and LEF in aging populations. With respect to PA_AGES-I_, increased physical activity consistently incurred better LEF performance. However, while all LEF regression models were highly significant, the variance captured by each model differed widely according to LEF task. In this regard, results were consistently strongest in STR models and weakest with TUG. This may be due to isometric strength tests having less propensity for influence from external modifying factors and inter-rater variability, as reflected by conflicting discourse on the comparative reliability of gait speed measurement and TUG^42^. Nevertheless, a comparative assessment of each LEF task and its relationship with physical activity is beyond the scope of the present work, but future investigation in this regard could help to clarify these findings.

The identification of ${N}_{m}$ and ${\mu }_{m}$ as significant partial mediators of this relationship is strong evidence that physical activity promotes mobility in aging through the preservation of skeletal muscle quantity and quality. The directionality of this relationship is evidenced by the signs of β coefficients for ${N}_{m}$ and ${\mu }_{m}$ in each of the present regression models. In relation to PA_AGES-I_, increased physical activity incurred larger lean muscle distributions with mean radiodensities further from 0 HU, conferring denser, healthier lean tissue. This relationship was likewise indicated in prospective models of LEF_AGES-II_, which consistently found that improved performance across all LEF tasks was related to higher ${N}_{m}$ and ${\mu }_{m}$ values.

In this regard, it is important to further outline how these NTRA parameters are associated with body composition. As noted, the NTRA method takes advantage of standard mathematical parameters that define the Gaussian distributions of radiodensity values from three canonical soft tissue types: adipose tissue, loose connective or water-equivalent tissue, and lean muscle. This procedure generates eleven subject-specific NTRA parameters that altogether serve as a multivariate quantitative construct for capturing changes in soft tissue quantity and lean tissue quality. These parameters can be grouped by both their relationship with soft tissue and their mathematical origin; for example, the amplitude parameter, $N$, is linked with soft tissue quantity through its direct association with the count of voxel elements for each tissue type. In contrast, greater lean tissue quality, reflected by limited partial volume effects from characteristic myosteatosis^38^, is indicated by narrower (↓$\sigma$), less-skewed (↓$\alpha$) muscle peaks located further from 0 HU (↑$\mu$). To our knowledge, this is first time that changes in soft tissue radiodensity have been shown to mediate the relationship between self-reported PA and LEF, and these findings are especially robust given both the prospective nature of this work and the large sample size offered by AGES-Reykjavík.

Another key advantage of the present NTRA-based methodology stems from its derivation from CT images. As an imaging modality widely reported for its standardizability in diagnostic applications and pathophysiological monitoring^43,44^, CT-derived distributions of soft-tissue radiodensity can be directly compared across clinical contexts. As such, NTRA-based classification is a highly reproducible method for assessing changed in body composition that can be readily built into existing CT analysis frameworks. This could have numerous implications for the field of aging research, which typically relies on estimates of body composition, such as BMI. For example, aging-related changes in BMI have been associated with cognitive aging; Arvanitakis et al. found that higher BMI in late-life was protective against cognitive decline, likely due to less sarcopenic muscle wasting^12^. In contrast, the recent work of Kivimaki et al. shows that midlife obesity is tied to cognitive decline and an increased risk for developing dementia^10^. These age-related differences in the impact of body composition on cognitive health are problematic and cannot be resolved using traditional anthropometric estimates of body composition like BMI—a notion which further motivates the pursuit of novel soft tissue assessment methods.

Nevertheless, assessment of the present findings necessitates the consideration of several key study limitations. Firstly, while both NTRA and LEF parameters were repeated measures available from both AGES timepoints, self-reported physical activity was only reported at AGES-I. As such, the present study cannot conclude whether the observed decline in LEF measures is associated with potential changes in physical activity levels. There were also a limited number of covariates considered for this work; in addition to age, sex, BMI, and LEF_AGES-I_ it may have been of interest to test other comorbidities, biometrics, or psychosocial factors in our models^45^. Likewise, as the AGES-Reykjavík study was almost entirely comprised of Caucasian subjects^46^, these findings may not be generalizable to other races or ethnicities.

Finally, in order to further generalize the NTRA method as a complimentary tool for studies involving physical activity assessment in elderly individuals, it is essential to discuss existing measurement constructs for physical activity and some of their potential limitations. Questionnaires, such as the one utilized in AGES-Reykjavík, have been validated for estimating physical activity in community health research at the population-level^47,48^. However, self-reported measures have shown limited reliability and validity compared to objective methods of physical activity measurement, such as accelerometry^49^. Nevertheless, systematic review findings report significant data heterogeneity from accelerometer-based studies due to differences in accelerometer design^50^ and disagreement on accelerometer cut-points for certain types of physical activity^51^. Furthermore, wearing accelerometers and similar devices could artificially motivate study participants to perform more activity than usual^49^. However, self-reporting can also be vulnerable to measurement bias from social desirability or faulty recall^52^, which may be particularly problematic in elderly subjects with significant memory limitations^53^. Older individuals are likewise more likely to engage in light- to moderate-intensity physical activity, which are generally the most difficult types of activity to recall accurately^54^. As such, exploring alternative constructs for measuring the impact of physical activity on skeletal muscle health could further situate its role in healthy aging while clarifying the underlying pathophysiological mechanisms of sarcopenia. This study has shown the potential of NTRA-based soft tissue radiodensity analysis in this regard.

## Materials and methods

### The AGES-I and AGES-II databases

The AGES-Reykjavík study began in 2002 with the recruitment of 3316 healthy volunteer subjects between 66 and 98 years of age (mean: 77.46) to participate in a series of two multimetric health assessments separated by approximately 5 years: AGES-I and AGES-II^29,55^. Written and informed consent was obtained from all participants, and ethical approval for patient data acquisition was obtained by the Icelandic Science and Ethics Committee (RU Code of Ethics, cf. Paragraph 3 in Article 2 of the Higher Education Institution Act no. 63/2006). All present methods were carried out in accordance with the relevant guidelines and regulations outlined in this approval. In addition to receiving CT scans (see “CT acquisition and segmentation”) and a battery of four lower extremity function (LEF) tests (see “LEF biometrics measurement”), subjects were tasked with self-reporting their level of moderate to vigorous physical activity in the past 12-months (PA_AGES-I_) using a five-point Likert scale ranging from 1 (‘Never’) to 5 (‘High’). Of the full AGES-Reykjavík recruitment, n = 3157 subjects in AGES-I had complete datasets for the purpose of this work, and n = 3098 of these subjects remained full participants in AGES-II. Summary statistics from AGES-I and AGES-II datasets were assembled for the present study (see Table 1).

### LEF biometrics measurement

A battery of four LEF tasks were assessed as part of the AGES-Reykjavik study as previously described^29,55,56^: normal and fastest-comfortable gait speed (GSN and GSF, respectively), timed up-and-go (TUG), and isometric leg strength (STR). A minimum of two LEF test leaders were tasked with recording these biometrics, and all study personnel were trained on requisite measurement procedures. Subjects were provided with detailed instructions on each LEF task to ensure the reliability of recorded data.

Gait measurement involved participants walking a straight-line distance of 6 m in accordance with established protocol^57^, and speed was recorded in meters per second (m/s) over two trials, which were averaged together for each subject. Individuals who could not complete these two trials were excluded from analyses.

The TUG test measured the time (in seconds) each subject required to stand from a seated position (height = 45.5 cm^58^), walk three meters, turn around, walk back to the chair, and sit down again^56^. Those who could not rise from the chair by themselves or walk unaided were excluded from the test, and the duration of each subject’s first complete trial was recorded for analyses.

Finally, STR was measured in Newtons (N) via knee extension using an adjustable digital dynamometer on a fixed chair (Good Strength, Metitur, Palokka. Finland)^59^. Here, subjects’ ankles were fastened to a strain-gauge transducer to record maximal sustained force, and all trials utilized a fixed knee angle of 60° from full extension; each subject performed two 4-s trials separated by a 30-s rest period, and STR was selected as the greater of the two sustained forces in these trials. Participants who had received surgery on their leg or hand were excluded from the tests, as well as individuals diagnosed with any ischemic heart condition two months prior to the study.

### Physical activity and covariate assessment

Physical activity was assessed in AGES-I by a self-reported questionnaire; here, subjects were asked to report their weekly participation in moderate–vigorous intensity physical activity over the previous year. Provided examples of activities that qualified as moderate–vigorous in intensity included badminton, golf, biking, swimming, heavy gardening, weightlifting, hiking/mountain climbing, fast walking/heavy housework, rowing, aerobics, jogging, and running^57^. Predefined physical activity categories were presented on a five-level Likert scale as: (1) never, (2) rarely, (3) occasionally (weekly, but $<$ 1 h), (4) moderate (1–3 h per week), and (5) high ($>$ 4 h per week). Age, sex, and body mass index (BMI) were assessed in AGES-I and AGES-II and selected as covariates for the purpose of the present work; age and sex were assessed by questionnaire, while BMI was calculated by dividing body weight, measured in kilograms (kg), by height squared, measured in meters (m^2^), in accordance with established practice^60^. Finally, baseline LEF performance obtained at AGES-I (LEF_AGES-I_) was likewise included in all LEF_AGES-II_ models as a covariate to adjust for the possibility that a prospective LEF association may be partially explained by the cross-sectional association between PA and LEF recorded during AGES-I.

### CT acquisition and segmentation

All AGES-Reykjavík participants were scanned with a 4-row CT detector system (Sensation; Siemens Medical Systems, Erlangen, Germany) using a standardized helical scanning protocol (120 kVp, 140 mAs, 1 mm thickness, Pitch = 1, and 4 mm collimation), as previously described^61^. Standard calibration and daily quality assurance procedures were performed by multiple CT technicians to ensure the reliability and validity of acquired CT data. The mean effective dose of radiation to participants was estimated at 1.61 mSv. Localized scanning regions extended from the iliac crest to the knee joint, and prior to transaxial imaging, correct scanning positions were determined by utilizing the maximum femoral length on an anterior–posterior localizer image followed by femoral long axis localization. Following each CT image acquisition, a single 10 mm cross-section was taken from the mid-thigh of each subject, located halfway between the hip acetabulum and the knee joint. Within this scanning field-of-view, a density calibration phantom (Image Analysis, Columbia, KY, USA) was included for all subject to allow for the linear transformation of pixel attenuation coefficients to their equivalent radiodensity values. This transformation resulted in the definition of subject-specific radiodensitometric distributions across the soft tissue range of − 200 to 200 HU, as described^38^. Subjects with missing or incomplete CT data were excluded from analyses.

### Nonlinear trimodal regression analysis (NTRA)

The computational method utilized to model radiodensity distributions featured a form of modified nonlinear regression analysis where the distribution of radioabsorption values (measured in HU) across voxel elements is taken to be a quasi-probability density function defined by three summed Gaussian distributions:

$${\sum }_{i=1}^{3}\varphi \left(x,{N}_{i},{\mu }_{i},{\sigma }_{i},{\alpha }_{i}\right)={\sum }_{1}^{3}\frac{{N}_{i}}{{\sigma }_{i}\sqrt{2\pi }}{e}^{-\frac{{(x-{\mu }_{i})}^{2}}{2{{\sigma }_{i}}^{2}}}erfc\left(\frac{{\alpha }_{i}(x-{\mu }_{i})}{{\sigma }_{i}\sqrt{2}}\right),$$

where $N$ is the amplitude, $\mu$ is the location, $\sigma$ is the width, and $\alpha$ is the skewness of each distribution—all of which are iteratively evaluated at each radiodensity bin, $x$. This trimodal definition operationalizes the hypothesis that HU distributions across segmented soft tissue represent the sum of three distinct tissue types whose linear attenuation coefficients occupy approximate HU domains: fat [− 200 to − 10 HU], loose connective, water-equivalent tissue and atrophic muscle [− 9 to 40 HU], and lean muscle [41–200 HU]. The respective inwardly-sloping asymmetries of fat and muscle distributions are captured by their positive and negative skewness terms, while the central ‘connective’ tissue distribution is assumed to be standard, or non-skewed. Next, to estimate radiodensity distribution parameters, an iterative curve-fitting procedure featuring a generalized reduced gradient algorithm was employed to minimize the sum of standard errors at bin. This general procedure, cited as the ‘NTRA’ method^37^, generates eleven subject-specific ‘NTRA parameters’ as a multivariate quantitative construct for capturing changes in soft tissue quantity and lean tissue quality.

### Multivariate multiple regression models and statistical analyses

To interrogate the mediating role of soft tissue NTRA parameters on the relationship between physical activity and LEF performance, we fitted a series of multivariate multiple regression models on 5-year longitudinal data captured between AGES-I and AGES-II timepoints. These models were adjusted for age, sex, BMI, and baseline LEF_AGES-I_ as relevant covariates, and to account for the simultaneous performance of multiple hypothesis tests, HB correction for multiple statistical comparisons was employed^62^, assuming a target ${\alpha }_{T}= 0.05$. Between-dataset differences in covariates, LEF, and NTRA parameters were likewise assembled as summary statistics, and significant cohort differences were tested using paired sample $t$ tests. Significance was set at $p<$ 0.05 (adjusted for multiple comparisons) in all statistical analyses.

### NTRA parameter mediation analysis on the relationship between PA_AGES-I_ and LEF_AGES-II_

**Figure 2:**
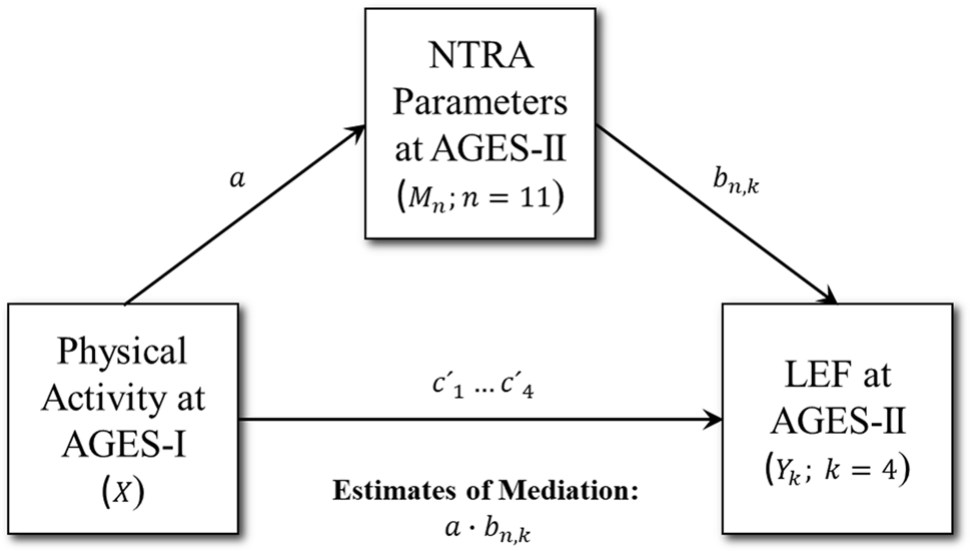
Conceptual path diagram for the present NTRA-based multiple mediator model, showing the theorized role of soft tissue radiodensity changes (characterized by eleven NTRA parameters) on the longitudinal relationship between physical activity and our four LEF tasks.

The R software package ‘RMediation’ was employed for the present mediation analysis^63^, which followed a standard prospective procedure in accordance with the conceptual mediation model depicted in Fig. 2. The first step in this procedure involved modelling the relationship between PA_AGES-I_ and LEF_AGES-II_. Next, the relationship between PA_AGES-I_ and all eleven NTRA_AGES-II_ parameters was modelled, and significant parameters from these models, adjusted for Type I errors, were then tested as independent predictors of LEF_AGES-II_. Indirect mediation effects from these NTRA parameters were then calculated, along with any direct effects from PA_AGES-I_ on LEF_AGES-II_. This was performed by first testing the attenuation of PA_AGES-I_ β coefficients in our prior PA_AGES-I_ → LEF_AGES-II_ regression models following the addition of implicated NTRA mediators. Finally, a bootstrapping procedure using the Hayes and Preacher SPSS macro^64^ was performed to interrogate the significance of indirect and direct effects.

## Data Availability

The AGES I-II dataset cannot be made publicly available, since the informed consent signed by the participants prohibits data sharing on an individual level, as outlined by the study approval by the Icelandic National Bioethics Committee. Requests for these data may be sent to the AGES-Reykjavik Study Executive Committee, contact: Ms. Camilla Kritjansdottir, Camilla@hjarta.is.
